# Hepatitis E Virus Prevalence among Blood Donors, Ouagadougou, Burkina Faso

**DOI:** 10.3201/eid2204.151728

**Published:** 2016-04

**Authors:** Kuan A. Traoré, Jean Bienvenue Ouoba, Hortense Rouamba, Yacouba K. Nébié, Honorine Dahourou, Frédéric Rossetto, Alfred S. Traoré, Nicolas Barro, Pierre Roques

**Affiliations:** Author affiliations: Université de Ouagadougou, Ouagadougou, Burkina Faso (K.A. Traoré, J.B. Ouoba, A.S. Traoré, N. Barro);; Commissariat à l’Energie Atomique , Service d’Immuno-Virologie, Fontenay-aux-Roses, France (K.A. Traoré, F. Rossetto, P. Roques);; INSERM, U1184, Fontenay-aux-Roses (K.A. Traoré, F. Rossetto, P. Roques);; Université Paris-Sud XI, Orsay, France (K.A. Traoré, F. Rossetto, P. Roques);; Centre Médical de Samandin, Ouagadougou (H. Rouamba);; Centre National de Transfusion Sanguine, Ouagadougou (Y.K. Nébié, H. Dahourou)

**Keywords:** IgG, IgM, hepatitis E, HEV, anti-HEV, viruses, blood transfusion, foodborne, waterborne, bloodborne, travel-related, Ouagadougou, Burkina Faso, West Africa

**To the Editor:** The safety of blood product use is continually improving, but blood transfusion remains a challenge in Africa, given the high prevalence of bloodborne pathogens (*1*). In Africa, the main serologic tests done to reduce blood transfusion risks are for HIV and hepatitis B and C viruses. However, unknown or emerging pathogens among the population of blood donors, such as hepatitis E virus (HEV), may also jeopardize transfusion safety. 

HEV is emerging as a potential threat to blood safety. High rates of HEV IgG prevalence among blood donors have been found in studies in the United States (7.7%), England (13.5%), France (16.6%), and Spain (19.6%) (*2*,*3*). A study in Iran showed a prevalence of 14.3% (*4*), and a study in China showed rates of up to 22.7% (*5*). Cases of HEV transmission by transfusion or transplantation have been reported, and recent studies in France and England showed risk for HEV in donated blood ranging from 1/2,218 to 1/2,848 donations (*5*,*6*).

 In Burkina Faso, the prevalence of HEV IgG has been reported as 11.6% among pregnant women during 2012. Prevalence is >70% among butchers, who form a population exposed to pigs, which are a reservoir for HEV (*7*,*8*). To determine whether HEV continues to circulate among human populations outside known at-risk populations, we investigated prevalence of HEV IgG and IgM in the blood donor population of Ouagadougou.

During June and July 2014, we recruited 1,497 first-time blood donors (398 women, 1,099 men) within the National Blood Transfusion Centre in Ouagadougou. Persons 17–65 years of age who weighed >50 kg were included (Figure, panel A). Candidate donors were excluded if they had previously received blood transfusions, had jaundice or clinical signs of hepatitis, were pregnant, or had sexual contact with multiple partners. Demographic data collection was limited to age and sex, and residual serum specimens were anonymized as approved by the Ethics Committee of the National Blood Transfusion Centre. We used Dia.Pro IgG ELISA (Diagnostic Bioprobe Srl, Sesto San Giovanni, Italy) to detect HEV IgG; this assay uses HEV-specific synthetic antigens derived from open reading frame (ORF) 2 and ORF3 of all 4 HEV subtypes. We used Wantai ELISA (Wantai Biologic Pharmacy Enterprise Co., Ltd., Bejiing, China) to test 92 randomly selected samples for HEV IgG, which showed concordant results (data not shown) (*8*). We also used the Wantai ELISA for the detection of HEV IgM; this test has a sensitivity of 97.1% (95% CI 94.6%–98.5%) and a specificity ranging from 95.3% in serum samples from patients with acute hepatitis A to 100% in healthy donors (http://www.ystwt.cn/IFU/HEV/HEV-IgM_CE.pdf). The HEV IgM positive samples were tested twice for accuracy. All tests were performed according to the manufacturers’ instructions; positive and negative controls were used in each plate.

**Figure:**
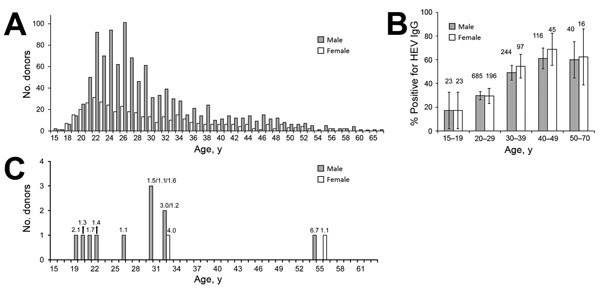
Age and sex distributions and HEV test results for blood donor population, Ouagadougou, Burkina Faso, 2014. A) All blood donors. Women: mean age 29.62 y, median 27 y, range 17–58 y; men: mean age 29.86 y, median 27 y, range 15–70 y. B) Donors whose samples were positive for HEV IgG. Numbers above bars indicate number of donors tested. Error bars indicate 95% CI for percentage in each category. C) Age and sex distribution of blood donors whose serum samples were positive for HEV IgM. Numbers on data bars are ratios of specific ELISA optical density to cutoff values (IgM index); ratios ≥1 are considered positive. Ratios are shown for each donor. HEV, hepatitis E virus.

The prevalence of HEV IgG was 39.0% (95% CI 36.5%–41.5%) by using Dia.Pro ELISA (Figure, panel B). This prevalence was twice that found in 2012 (*8*), but such wide variations were commonly found in Africa (*9*). In France, prevalence ranged widely, from 10% in the north to 52% in the south (*6*). HEV IgG prevalence increased significantly with age (p<0.001 by χ^2^ test for trend) in both male and female donors, but age variation explained only partially the differences in the study population and those from a previous study (*8*). As described in France and other high-income countries (*4*,*6*), Traoré et al. found HEV genotype 3 in swine in Burkina Faso (*10*); thus, poor sanitation that disperses this oral–fecal transmitted virus might result in a high prevalence of HEV antibodies among the general population without causing epidemic illness that is more often associated to genotype 1.

Using the Wantai test, we found HEV IgM, a marker of recent infection, in samples from 2 women and 11 men in the blood donor population (1.9%, 95% CI 1.2–2.6% [Figure, panel C]). Samples from 7 men were positive for HEV IgG.

The HEV exposure prevalence we observed is similar to most of the published data from countries reporting endemic HEV and silent infection (*6**,**7*). IgM seroprevalence of 1.9% is indicative of low ongoing infection cycles, although no reference test is available (*2*). Our study was limited by the absence of HEV RNA screening to assess the presence of HEV particles and genotype in donated blood. However, HEV circulation is supported by 1) IgM signs of recent infection; 2) the commonality of silent infections with HEV, specifically genotype 3; and 3) another study that showed a clear, although rare, positive relationship between the number of IgM-positive samples and the number of HEV RNA-positive samples (*4*).

The risk for HEV infection through transfusions of donated blood emerged in West Africa in a similar way as described in European countries. Further assessment of the transfusion risk associated with HEV-positive donors will require an evaluation of HEV RNA in prospective donors and posttransfusion surveillance of occurrence of hepatitis.
